# ‘Dopamine-first’ mechanism enables the rational engineering of the norcoclaurine synthase aldehyde activity profile

**DOI:** 10.1111/febs.13208

**Published:** 2015-02-09

**Authors:** Benjamin R Lichman, Markus C Gershater, Eleanor D Lamming, Thomas Pesnot, Altin Sula, Nicholas H Keep, Helen C Hailes, John M Ward

**Affiliations:** 1 Department of Biochemical Engineering, University College LondonUK; 2 Department of Chemistry, University College LondonUK; 3 Crystallography, Biological Sciences, Institute for Structural and Molecular Biology, Birkbeck, University of LondonUK

**Keywords:** alkaloid biosynthesis, biocatalysis, enzyme engineering, enzyme kinetics, enzyme mechanism

## Abstract

Norcoclaurine synthase (NCS) (EC 4.2.1.78) catalyzes the Pictet–Spengler condensation of dopamine and an aldehyde, forming a substituted (*S*)-tetrahydroisoquinoline, a pharmaceutically important moiety. This unique activity has led to NCS being used for both *in vitro* biocatalysis and *in vivo* recombinant metabolism. Future engineering of NCS activity to enable the synthesis of diverse tetrahydroisoquinolines is dependent on an understanding of the NCS mechanism and kinetics. We assess two proposed mechanisms for NCS activity: (a) one based on the *holo* X-ray crystal structure and (b) the ‘dopamine-first’ mechanism based on computational docking. *Thalictrum flavum* NCS variant activities support the dopamine-first mechanism. Suppression of the non-enzymatic background reaction reveals novel kinetic parameters for NCS, showing it to act with low catalytic efficiency. This kinetic behaviour can account for the ineffectiveness of recombinant NCS in *in vivo* systems, and also suggests NCS may have an *in planta* role as a metabolic gatekeeper. The amino acid substitution L76A, situated in the proposed aldehyde binding site, results in the alteration of the enzyme's aldehyde activity profile. This both verifies the dopamine-first mechanism and demonstrates the potential for the rational engineering of NCS activity.

## Introduction

**Fig. 1:**
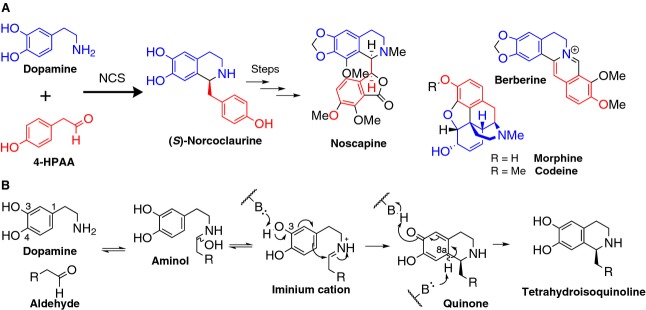
The NCS Pictet–Spengler reaction. (A) The role of NCS in the BIA pathway. Dopamine and 4-HPAA react via a Pictet–Spengler condensation, catalyzed by NCS, to form (*S*)-norcoclaurine. The colours of atoms show their origin: blue atoms derive from dopamine and red from 4-HPAA. (B) Overall mechanism of tetrahydroisoquinoline Pictet–Spengler reaction. NCS reaction mechanism as proposed previously [9]. A similar mechanism is postulated for the phosphate catalysed reaction, giving a racemic product [14]. The reaction is initiated by formation of an aminol, followed by elimination of water to form an iminium cation. The subsequent rate-limiting step, deprotonation of the dopamine 3-hydroxy, triggers cyclization onto the iminium. The final, irreversible, step is deprotonation of the quinone 8a-H to form norcoclaurine.

In angiosperms, the enzyme norcoclaurine synthase (NCS) (EC 4.2.1.78) is responsible for the formation of (*S*)-norcoclaurine via a Pictet–Spengler condensation of the tyrosine derivatives dopamine and 4-hydroxyphenylacetaldehyde (4-HPAA) (Fig. 1) [1,2]. (*S*)-Norcoclaurine is the precursor to all benzylisoquinoline alkaloids (BIAs), a diverse group of ∼ 2500 natural products [2]. A number of BIAs have significant biological activities, notably the analgesics morphine and codeine, the antibiotic berberine, and the anticancer candidate noscapine [3,4].

NCSs from *Thalictrum flavum* (*Tf*NCS) and *Coptis japonica* (*Cj*NCS2) were shown to have a broad aldehyde substrate scope, enabling the enzymatic formation of diverse (*S*)-tetrahydroisoquinolines (THIQs) [5–8]. Both enzymes accept most phenylacetaldehydes but do not turn over α-substituted aldehydes. Particularly notable is the turnover of the linear aliphatic aldehydes by *Cj*NCS2 and the large 1-napthylacetaldehyde by *Tf*NCS because these substrates have properties that are very different from those of the natural substrate 4-HPAA. By contrast to the aldehyde, little variation in dopamine structure is tolerated: the 3-hydroxy moiety is crucial for successful turnover [6,9].

Progress in the field of synthetic biology has led to the development of a number of microbial BIA pathways which include recombinant NCS. However, it is not clear whether NCS has activity in any of these *in vivo* settings as a result of the presence of a high non-enzymatic background Pictet–Spengler reaction [10,11]. In some of these synthetic pathways, chirality is established in the final products courtesy of the selectivity of enzymes downstream of NCS [12]. The apparent ineffectiveness of recombinant NCS *in vivo* contrasts with its natural *in planta* behaviour, where its removal results in significant reduction of alkaloid production [13]. The non-enzymatic background reaction observed in the recombinant systems is likely to be caused by phosphates: inorganic phosphate is capable of catalyzing the Pictet–Spengler reaction between dopamine and aldehydes, forming racemic THIQs [14]. Consequently, the use of phosphate buffer has adversely affected a number of *in vitro* investigations into the NCS mechanism and kinetics [1,9,15–17].

**Fig. 2:**
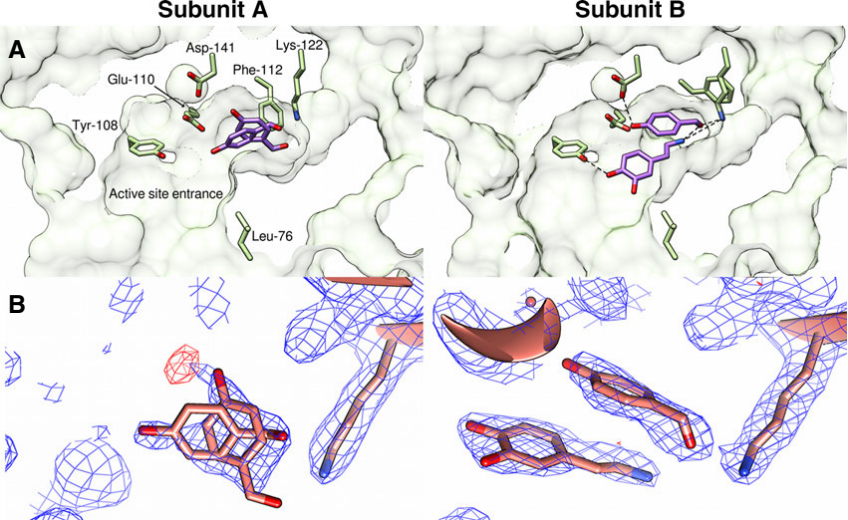
*Holo Tf*NCS crystal structure (2VQ5). (A) Cross-sections of NCS subunits showing active site ligands and residues. Sections of the protein solvent accessible surface are shown and selected active site residues are represented by yellow sticks. Ligands are represented by purple sticks. Note the different position of Phe112 in the active sites (for examination of the crystal packing interaction and Phe112 conformations, see Figs 5 and 6). (B) Electron density map (blue, 2*F*_o_ − *F*_c_, contoured at 1σ) and difference map (red, 2*mF*_o_ − *DF*_c_, contoured at 3σ) of 2VQ5, with Lys122 and ligands represented by sticks. Note that, in both subunits, the density does not fit around certain ligand atoms. Images generated using *Chimera*.

**Fig. 3:**
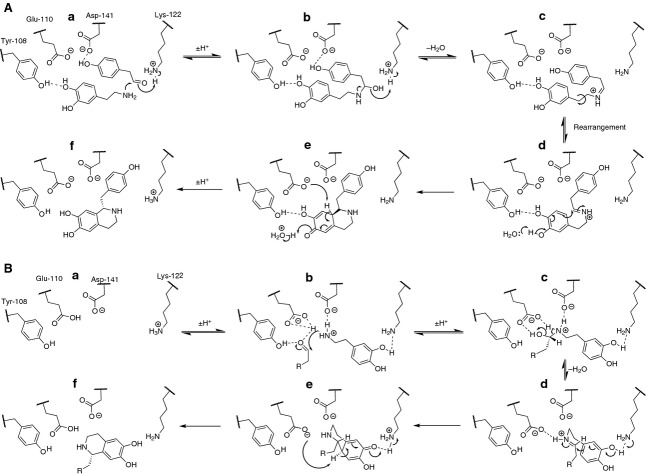
NCS catalytic mechanisms. (A) HPAA-first mechanism. Mechanism proposed on the basis of ligand binding modes observed in crystal structure. (a) Ligands are arranged as observed in the subunit B of 2VQ5. 4-HPAA is deepest in the active site, bound to Lys122. Dopamine attacks the activated aldehyde. (b) Loss of water from the aminol catalyzed by Lys122. (c) Iminium cation rearranges in active site through rotation and *cis-trans* isomerization. (d) Cyclization onto iminium occurs, aided by Tyr108. (e) Concurrently, Glu110 removes the proton from the quinone to form the product (f). Scheme produced in accordance with previous studies [16,18]. (B) Dopamine-first mechanism. Mechanism proposed on the basis of computation docking (Fig. 4). (a) Active site without ligands, mechanistically important residues represented. (b) Dopamine binds to Lys122 via the catechol and to the carboxylic acid residues via the charged nitrogen. Aldehyde binds to Glu110 and Tyr108 and is attacked by dopamine. (c). The (*S*)-aminol is formed. Loss of water is mediated primarily by Glu110 and aided by Asp141. (d) Enantioselective cyclization of the iminium cation occurs, catalysed by the basic Lys122. (e) The quinone intermediate is deprotonated by Glu110 resulting in formation of the product (f). For computational docking and p*K*_a_ predictions that aided the development of this mechanism, see Fig. 4 and Table 3 respectively.

**Fig. 4:**
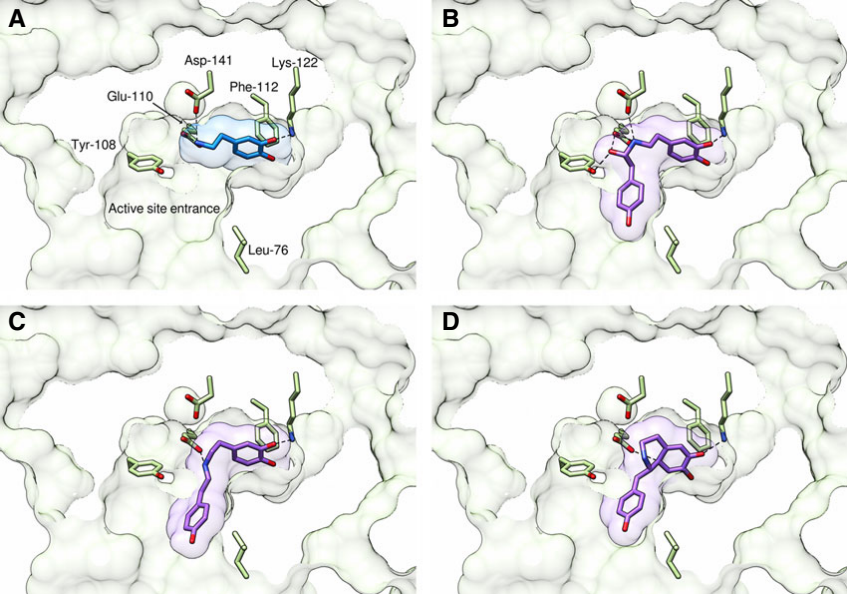
Predicted conformations of reaction intermediates in NCS active site. Reaction intermediates (Fig. 7) were docked in the NCS active site (subunit A, 2VQ5) using autodock vina [31]. The highest ranked conformations are shown for (A) dopamine, (B) (*S*)*-*aminol, (C) *trans-*iminium cation and (D) *anti*-quinone. The *cis*-iminium binds in a similar manner to the *trans* but is in a conformation that would lead to wrong product enantiomer. Black dotted lines show predicted electrostatic and catalytic interactions. Active site residues carbons are coloured yellow; docked ligands carbons are coloured purple. Ligand accessible surfaces are represented by transparent purple. A section of the protein solvent accessible surface is represented in green. More detailed information on protein–ligand interactions is provided in Table 1. Images generated using *Chimera*.

Solution of the *Tf*NCS crystal structure enabled the identification of the enzyme active site residues (Fig. 2A) [16]. An enzyme mechanism for the natural substrates was developed based on the *holo* crystal structure containing dopamine and a nonproductive aldehyde (Fig. 3A) [18]. This mechanism involves the binding of 4-HPAA to the enzyme prior to dopamine (the ‘HPAA-first’ mechanism). The mechanism features the aldehyde buried in the active site, and so does not appear to account for the aldehyde promiscuity of the enzyme. Subsequently, an alternative mechanism was developed based on computational docking, in which dopamine binds to the enzyme prior to 4-HPAA (the ‘dopamine-first’ mechanism) (Figs 3B and 4) [6].

A clearer understanding of the mechanism and kinetics of NCS is required not only to understand the early steps of BIA biosynthesis, but also to further develop NCS as a tool for both synthetic biology and biocatalysis. This knowledge will enable the future rational engineering of the enzyme, leading towards the optimization of the recombinant enzyme for *in vivo* activity, and also the broadening of the enzyme's substrate scope.

In the present study, we reassess the mechanism and kinetics of NCS; this enables us to rationally engineer the activity of the enzyme, increasing its activity towards an unnatural substrate. First, we compare the two proposed mechanisms: the HPAA-first mechanism, based on the *holo* X-ray crystal structure, and the dopamine-first mechanism, based on computation docking. Amino acid substitutions are used to probe the role of active site amino acids; their activities support the dopamine-first mechanism. Suppression of the non-enzymatic background chemical reaction reveals new kinetic parameters for NCS, showing it to be a catalytically inefficient enzyme with remarkably high apparent *K*_m_ values. Finally, the substitution L76A in the aldehyde binding site proposed by the dopamine-first mechanism results in the modification of the enzyme's aldehyde activity profile.

## Results and Discussion

### The *holo* crystal structure provides ambiguous data

The HPAA-first mechanism was based on the observed ligand binding modes in the *holo* NCS crystal structure. This structure was obtained by soaking NCS crystals with solutions of dopamine and *para*-hydroxybenzaldehyde (PHB), an electron deficient non-productive 4-HPAA analogue. Subunit A of the *holo* crystal structure is shown to bind PHB in two different conformations, whereas subunit B binds both dopamine and PHB simultaneously (Fig. 2A) [16].

The ligands modelled into the active site of the *holo* crystal structure do not fit the electron density convincingly (Fig. 2B). The density observed in subunit A does not support the presence of two conformations of PHB, as modelled (Fig. 2B, subunit A). In subunit B, a methylene from dopamine does not fit in the density, and oxygen atoms from both ligands are in regions with no density (Fig. 2B, subunit B). Generally, the electron density appears to have insufficient definition to describe the identity of ligands present. This observed ambiguity may be a result of low occupancy, or perhaps a heterogeneous sample.

**Fig. 5:**
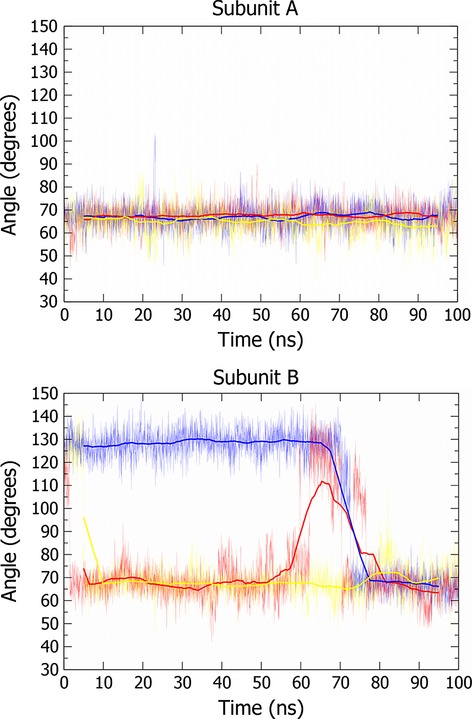
Conformation of residue Phe112 in molecular dynamics simulations. Simulations conducted in GROMACS with the charmm27 forcefield (see Materials and methods) [29,30]. Conformation of residue measured by recording angle between three atoms: the Cα of Phe112, the Cζ of Phe112 and the Cα of His106. The thin lines are the values for this angle; the thick lines are a moving average (0.5-ns window). Different colours represent independent simulations. Simulations of subunit A show that Phe112 is stable across 100 ns, fluctuating around 65–67°. Simulations of subunit B show that the Phe112 conformation around 125° is unstable and, in all simulations, it reverts to 65°. This suggests the conformation seen in subunit A is the most stable, and most common in solution.

The difference in the binding behaviour of the two subunits is likely to be a consequence of the different rotameric conformations of Phe112 (Fig. 2A). Evaluation of the crystal structures revealed that the Phe112 conformation present in subunit B is unusual; it is only present in 1% of phenylalanines in the Protein Data Bank [19]. This analysis is supported by molecular dynamics simulations suggesting that, in solution, the Phe112 conformation in subunit B reverts to that observed in subunit A (Fig. 5).

**Fig. 6:**
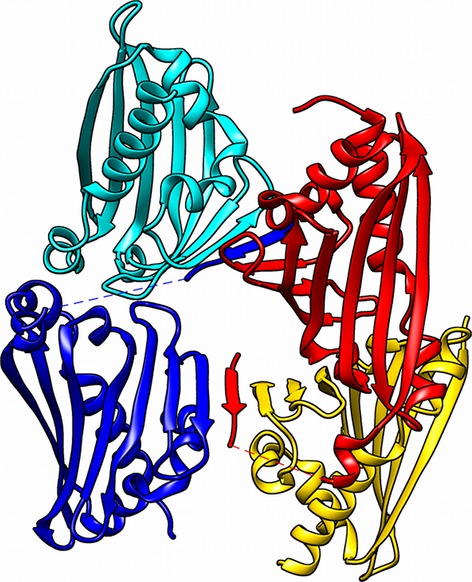
*Tf*NCS crystal structure packing. Cartoon representation of asymmetric unit in 2VQ5 [16]. Red and blue cartoons show subunit A. Cyan and yellow show subunit B. Note the subunit A N-terminal β-strand that causes the interaction between subunits A and B in the adjacent dimer. Image generated using *Chimera*.

The differences in the active sites of the subunits may be a knock-on effect from the tight crystal packing. The asymmetric dimer is held together by a non-native β-strand formed from the first nine N-terminal residues of subunit A in the adjacent dimer (Fig. 6). This N-terminal sequence is not necessary for enzyme activity [20,21] and is possibly part of a cleaved signal peptide (predicted by sosuisignal) [22].

Overall, the binding modes derived from the *holo* structure appear to be speculative on the basis of the observed electron density.

### Proposed HPAA-first mechanism cannot account for NCS activity

The HPAA-first mechanism was proposed based on the arrangement of the substrates in subunit B (Fig. 3A) [16,18]. A major drawback of the HPAA-first mechanism is that it cannot account for NCS reacting with a wide variety of aldehydes. First, the proposed interaction between Asp141 and the phenol of 4-HPAA would not be possible with many aldehydes. Second, it is unlikely that the active site can incorporate large aldehydes (such as 1-napthylacetaldehyde or citronellal) and dopamine simultaneously in the proposed stacked formation.

In this mechanism, the intermediate imine undergoes a rearrangement which includes an iminium *cis*-*trans* isomerization and the rotation of the entire aldehyde R-group. This step is vital for the subsequent rate-determining cyclization and deprotonation, which establish the chirality of the product. However, there is no evidence for this reorganization: the *holo* crystal structure could only provide evidence for the initial arrangement of the substrates.

The chemical basis of the mechanism also has limitations. Water is not sufficiently basic (p*K*_b_ = −1.7) to deprotonate the 3-hydroxy of dopamine in the rate-determining step; a basic active site residue is required [23]. Finally, the hydrogen bond between the quinone and Tyr108 would not provide electrostatic stabilization to the intermediate, and cannot account for the successful reaction when the 4-hydroxy moiety is not present.

### Computational docking reveals dopamine-first mechanism

**Fig. 7:**
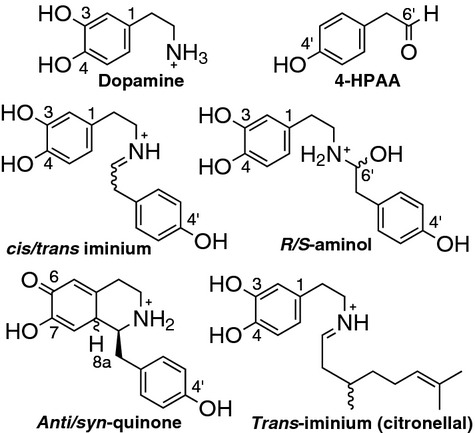
Structures of intermediates used in docking calculations. Structures and charges of the ligands used in computational docking experiments. Ligands were docked into the empty active site of the crystal structure of *Tf*NCS (2VQ5, subunit A) using autodock vina [16,31]. Both isomers of the aminol, iminium, quinone and iminium-citronellal intermediates were investigated: the wavy bonds show where the stereochemistry varied. For observed docking conformations, see Figure 4 and Table 1.

To explore alternative enzyme mechanisms, reaction intermediates were docked into the active site of *Tf*NCS (2VQ5, subunit A) (Fig. 7 and Table 1) [6]. This revealed binding modes that form the basis of the dopamine-first enzyme mechanism (Figs 3B and 4). Subunit A appears to be a better candidate for docking studies than subunit B on the basis of molecular dynamic simulations, which suggest the subunit A Phe112 conformation present is prevalent in solution (Fig. 5). The *holo* crystal structure 2VQ5 (with ligands removed) was used for computational docking because it has a superior resolution to the *apo* structure 2VNE.

**Table 1:** Binding modes calculated from *in silico* docking. Predicted binding affinity and protein-ligand interaction distances of docked reaction intermediates in the NCS active site (2VQ5, subunit A). Conformations predicted by autodock vina [31]. For full structures of intermediates and atom numbering information, see Fig. 7. Figure 4 shows the structures of selected binding modes. All binding modes described were the highest ranked by the software

Ligand	Binding affinity	Heteroatom distances Residues											Comments

		Tyr108		Glu110 (O1)^a^		Glu110 (O2)^a^			Lys122		Asp141		

		Atom on ligand^a^											

		N	6′O	N	6′O	N	6′O	8aH	3O	4′O	N	6′O	
	(kcal·mol^−1)^	Å											
Dopamine	−5.3	4.5		2.9					3.1		3.0		See Fig. 4A
4-HPAA	−5.2		4.4		3.0		3.4			3.1		3.4	4′O bound to Lys122^c^
*S*-aminol	−7.3	5.0	2.8	3.1	2.9				3.2		3.2	4.2	See Fig. 4B
*R*-aminol	−7.1	4.6	4.4	3.8	3.0	3.1			> 10	3.1	5.1	3.0	4′O bound to Lys122^c^
*cis*-iminium	−7.5	4.0		3.0		3.2			3.1		3.9		Similar to *trans*^d^
*trans*-iminium	−7.2	4.3		3.8		3.6			3.2		4.6		See Fig. 4C^d^
*S-*anti-quinone	−7	5.3		4.3				3.3	2.8		4.0		See Fig. 4D
*S-*syn-quinone	−6.8	8.3		> 10				9.6	> 10		> 10		Not in active site

a Both oxygen atoms on the Glu110 side chain are involved in docking interactions.

b For full structures of intermediates and their atom numbering, see Fig. 7.

c Close interactions between 4′O (from 4-HPAA) and Lys122 represent binding modes that do not fit with our proposed mechanism.

d The *trans-*iminium is in a conformation which would form the *S-*isomer in the subsequent cyclization step. The *cis* isomer conformation would lead to the incorrect *R* product.

A key factor in the biocatalytic utility of NCS is its ability to turn over a wide variety of structurally diverse aldehydes. Docking calculations provide a structural explanation for this observation: the most favourable binding modes for reaction intermediates show that only the aldehyde R-group is partially exposed to the solvent (Fig. 4 and Table 1). The poor tolerance of α-substituted aldehydes by NCS may be rationalized on steric grounds because the α-carbon is buried in the active site. Large aldehydes (but with no α-substitutions) may be accepted because the R-group bulk can be in the solvent, away from the enzyme.

### Behavior of variants support the dopamine-first mechanism

To probe the validity of the dopamine-first mechanism, the activities of particular NCS variants were investigated. The residues Tyr108, Glu110, Lys122 and Asp141 were selected for substitution based on both previous investigations and the *in silico* docking results [16]. Initially, two aldehydes, the natural substrate 4-HPAA and the aliphatic hexanal, were selected for investigation, aiming to probe whether any amino acid substitution affected the enzyme activity in a substrate-specific manner.

**Fig. 8:**
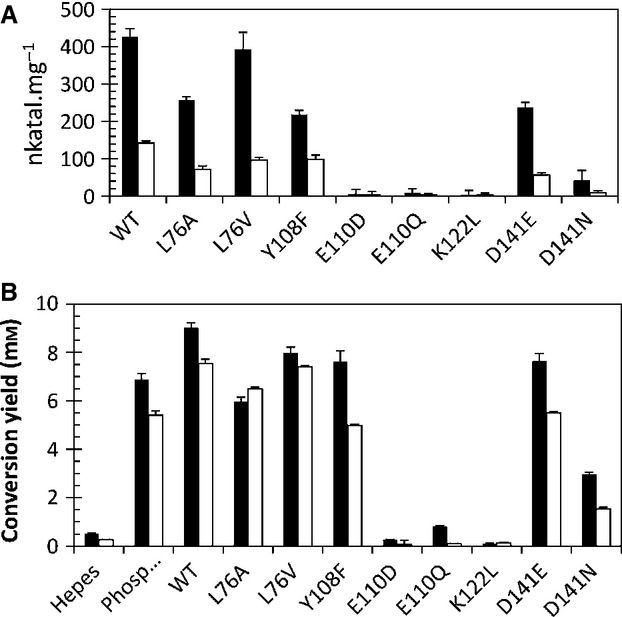
Behaviour of *Tf*NCS variants. (A) Enzyme activities. Initial rates of reactions between 10 mm dopamine and 2.5 mm aldehyde, catalysed by NCS variants. Aldehydes: 4-HPAA (solid bars) and hexanal (white bars). Values are the mean of three separate measurements, error bars indicate SDs. Assay methodology is as described in the Materials and methods. (B) Conversion yields of biotransformations. Enzymes and buffers were incubated with dopamine and aldehydes for 1 h at 37 °C. Both substrate concentrations were 10 mm, so that the maximum product concentration possible is 10 mm. Conversion yields were obtained by HPLC analysis and quantified by comparison with verified standards. Values are the means of three separate reactions, error bars indicate SDs. Products were also analyzed by chiral HPLC (Fig. 9). Enzyme melting points were measured showing all enzymes were folded in the assay conditions (Table 2).

**Fig. 9:**
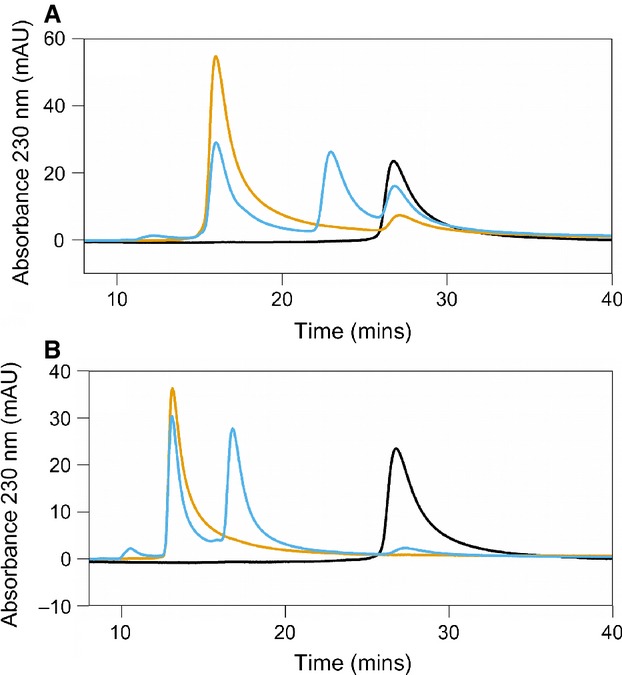
Chiral HPLC analysis of enzymatic reactions. (A) Dopamine and 4-HPAA. (B) Dopamine and hexanal. Dopamine standard (black trace) appears at 27 min. Phosphate buffer catalyzed reactions (blue trace) shows two equal sized peaks for the racemic products. Wild-type (WT) enzyme catalyzed reactions (orange trace) show a single isomer peak (> 95% *ee*). Chiral HPLC traces for mutants L76A, L76V, Y108F, D141E and D141N also demonstrated that reactions occurred with > 95% *ee* for both substrates. Mutants E110D, E110Q and K112L, and the reaction conducted in Hepes buffer, showed insufficient conversion to determine enantiomeric excess. Reaction conditions were as described in the Materials and methods, with substrate concentrations of 10 mm and 1-h reaction times. HPLC traces only shown starting from 10 min; prior to this, only the solvent front is visible.

All variants were judged to be folded under experimental conditions on the basis of their melting points (Table 2). All enzymes were more active with 4-HPAA than hexanal (Fig. 8). Enzymes, with sufficient activity, were observed to perform the reactions in Hepes buffer in a stereoselective manner [(*S*)*-*isomer, > 95% enantiomeric excess] (Fig. 9). The type of buffer used in the non-enzymatic reactions impacted greatly on the conversion. In phosphate buffer, conversions with 4-HPAA and hexanal were 69% and 54%, respectively (racemic product). By contrast, Hepes buffer reached only 5% and 3%, respectively (Fig. 8B).

**Table 2:** Melting and aggregation temperatures (°C) of *Tf*NCS variants. Data generated using an Avacta Optim instrument. Over a temperature ramp, *T*_m_ is calculated by observing changes in the intrinsic protein fluorescence spectrum; *T*_agg_ is calculated by changes in light scattering intensity. WT, wild-type

Protein	*T*_m_	*T*_agg_
WT	60.7 ± 0.1	60.8 ± 0.1
L76A	54.9 ± 0.1	54.4 ± 0.6
L76V	58.7 ± 0.1	59.1 ± 0.5
Y108F	61.5 ± 0.3	62.4 ± 0.4
E110D	57.2 ± 0.2	57.4 ± 0.5
E110Q	66.0 ± 0.4	65.8 ± 0.1
K122L	69.8 ± 0.3	67.9 ± 1.8
D141E	62.0 ± 0.3	61.8 ± 0.2
D141N	63.9 ± 0.1	62.8 ± 0.9

The variant K122L demonstrated no activity with 4-HPAA or hexanal, supporting the identity of Lys122 as the residue catalyzing the rate-determining cyclization step. Lys122 is able to catalyze this step because of its low p*K*_a_ (prediction ∼ 7.2), which is the result of burying a charge in a hydrophobic environment (Table 3). The stability increase of 9.1 °C conferred by the K122L substitution is a demonstration of the same effect (Table 2). The p*K*_a2_ phosphate, which is able to catalyse the racemic reaction, is also 7.2; this suggests that both Lys122 and phosphate catalyze the same step in the reaction mechanism [24].

**Table 3:** Active site residue p*K*_a_ predictions. Acid dissociation constants (p*K*_a_) of *Tf*NCS active site and reaction intermediate ionizable groups predicted by propka, version 3.1 [32]. The enzyme model was the X-ray structure 2VQ5, subunit A [16]. Ligand conformations were predicted by computational docking of reaction intermediates into the enzyme active site with autodock vina (Fig. 4 and Table^1^) [31]. These predictions contributed to the proposed mechanism (Fig. 3B)

	Tyr108	Glu110	Lys122	Asp141	Ligand N
Typical^a^	10.0	4.5	12.5	3.8	
Ligand^b^					
None	15.5	8.1	7.9	6.7	
Dopamine	13.3	5.7	7.2	4.1	15.8
*S*-aminol	15.2	5.6	7.2	4.2	13.6
*cis-*iminium	16.4	5.1	7.1	6.8	11.6
*trans-*iminium	16.2	5.4	7.1	7.0	11.4
*S-*anti-quinone	16.3	6.9	7.4	5.6	10.5

a Model p*K*_a_ values used by propka, version 3.1, at the start of calculations.

b Ligand structures are shown in Fig. 7. Docked structures are shown in Figure 4 and described in Table1.

Of the two carboxylic acid residues investigated, NCS variant activities show that only Glu110 is necessary for the reaction to proceed. That both a removal of charge (E110Q) and a change in charge position (E110D) eliminate activity supports the suggestion that Glu110 acts as a base, abstracting the quinone 8a-H (Fig. 3B, step e). On the other hand, the behaviours of Asp-141 variants suggest that the residue is not responsible for base catalysis, as, whilst D141N has 10% of WT activity, D141E retains 50% activity. This suggests that Asp141 may provide general electrostatic stabilization, rather than base catalysis.

Tyr108 appears to have a dual role: it not only contributes to the electrostatic properties of the active site, but also defines the shape of the cavity entrance (Fig. 4). This is reflected in a decrease in both rate and apparent *K*_m_ values in the Y108F variant (Table 4). The removal of the hydroxy moiety upon amino acid substitution opens up the space around the active site entrance, perhaps aiding aldehyde substrate binding. This change is more pronounced for 4-HPAA than hexanal, as the former has greater steric bulk.

**Table 4:** Apparent kinetic parameters for *Tf*NCS. Corresponding velocity/substrate concentration curves can be found in Fig. 10. The assay methodology is as described in the Materials and methods. WT, wild-type

Varied substrate	Constant substrate	Enzyme	Michaelis–Menten			

			*V*_max,app_	*k*_cat,app_	*K*_m,app_	*k*_cat,app_/*K*_m,app_
			(nkatal·mg^−1^)	(s^−1^)	(mm)	(s^−1^·mm^−1^)
Dopamine^a^	4-HPAA	WT	1150 ± 48	24 ± 1	22.2 ± 1.5	1.09 ± 0.08
		L76A	649 ± 22	13.6 ± 0.5	16.8 ± 1.0	0.81 ± 0.06
		Y108F	485 ± 13	10.2 ± 0.3	13.3 ± 0.7	0.76 ± 0.05
Dopamine^a^	Hexanal	WT	323 ± 7	6.8 ± 0.1	20.1 ± 0.7	0.34 ± 0.01
		L76A	196 ± 10	4.1 ± 0.2	17.0 ± 1.5	0.24 ± 0.02
		Y108F	221 ± 8	4.6 ± 0.2	16.9 ± 1.1	0.27 ± 0.02
4-HPAA^b^	Dopamine	WT	893 ± 43	18.7 ± 0.9	18.1 ± 1.4	1.03 ± 0.09
		L76A	682 ± 24	14.3 ± 0.5	17.4 ± 1.0	0.82 ± 0.05
		Y108F	219 ± 13	10.2 ± 0.3	13.3 ± 0.7	0.76 ± 0.05
Hexanal^b^	Dopamine	WT	205 ± 16	4.3 ± 0.3	13.4 ± 1.8	0.32 ± 0.05
		L76A	150 ± 5	3.2 ± 0.1	13.4 ± 0.8	0.24 ± 0.02
		Y108F	119 ± 7	2.5 ± 0.1	7.6 ± 0.9	0.33 ± 0.04
Dopamine^a^	(*S*)-citronellal	WT	153 ± 8	3.2 ± 0.2	13.8 ± 1.0	0.23 ± 0.02
		L76A	368 ± 19	7.7 ± 0.4	15.3 ± 1.0	0.50 ± 0.04
Dopamine^a^	(*R*)-citronellal	WT	115 ± 3	2.4 ± 0.1	10.3 ± 0.4	0.24 ± 0.01
		L76A	138 ± 5	2.9 ± 0.1	11.0 ± 0.6	0.26 ± 0.02
(*S*)-citronellal^b^	Dopamine	WT	22 ± 1	0.46 ± 0.02	0.38 ± 0.10	1.20 ± 0.31
		L76A	45 ± 2	0.93 ± 0.05	0.59 ± 0.09	1.57 ± 0.25
(*R*)-citronellal^b^	Dopamine	WT	17.9 ± 1.4	0.37 ± 0.03	0.53 ± 0.14	0.71 ± 0.20
		L76A	26.4 ± 1.6	0.55 ± 0.03	0.49 ± 0.11	1.13 ± 0.27

			Substrate inhibition^c^			
			
			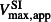	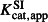	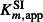	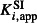
			(nkatal·mg^−1^)	(s^−1^)	(mm)	(mm)
(*S*)-citronellal^b^	Dopamine	WT	27.4 ± 1.8	0.57 ± 0.04	0.64 ± 0.12	37 ± 11
		L76A	64.1 ± 9.6	1.34 ± 0.20	1.06 ± 0.25	23 ± 11^d^
(*R*)-citronellal^b^	Dopamine	WT	33.9 ± 4.8	0.71 ± 0.10	1.42 ± 0.31	13 ± 4
		L76A	41.9 ± 6.6	0.88 ± 0.14	1.09 ± 0.29	15 ± 6^e^

a All kinetic parameters for dopamine were recorded with 2.5 mm aldehyde, and dopamine varied from 500 μm to 20 mm.

b All kinetic parameters for aldehydes were recorded with 2.5 mm dopamine, and aldehyde varied from 250 μm to 15 mm.

c Substrate inhibition equation: rate = *V*_max_ × [*S*]/(*K*_m_ + [*S*] + ([*S*]^2^/*K*_i_)).

d Calculated *P* > 0.05 for this coefficient (0.081).

e Calculated *P* > 0.05 for this coefficient (0.054).

### Kinetics reveals NCS operates with low catalytic efficiency

The use of phosphate buffer in previous kinetic experiments with NCS led to the generation of inaccurate kinetic parameters as a result of the non-enzymatic background reaction [1,6,9,15–17]. In this study, we performed all enzyme assays in Hepes buffer, in which there is far less non-enzymatic background (Fig. 8B). However, because of the increased background reaction rate at high substrate concentrations, kinetic parameters were determined using nonsaturating concentrations of the nonvaried substrates.

**Fig. 10:**
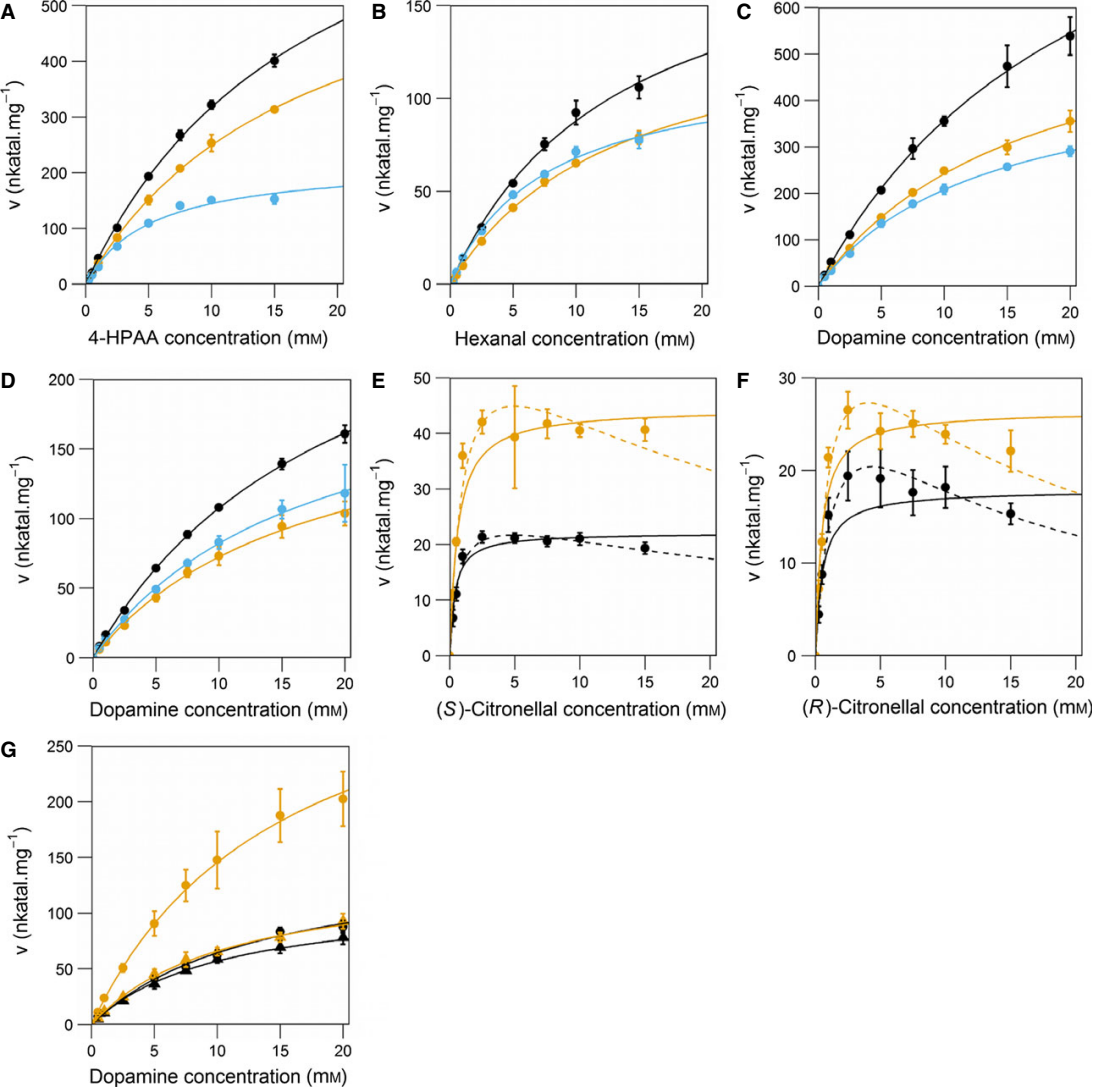
Velocity/substrate concentration curves for wild-type (WT, black), L76A (orange) and Y108F (blue). (A) Varying 4-HPAA concentrations with 2.5 mM dopamine. (B) Varying hexanal concentrations with 2.5 mM dopamine. (C) Varying dopamine concentrations with 2.5 mM 4-HPAA. (D) Varying dopamine concentrations with 2.5 mM hexanal. (E) Varying (*S*)-citronellal concentrations with 2.5 mM dopamine. (F) Varying (*R*)-citronellal concentrations with 2.5 mM dopamine. (G) Varying dopamine concentrations with 2.5 mM citronellal, circles show (*S*)-citronellal and triangles show (*R*)-citronellal. Nonlinear least-squares fits: solid lines Michaelis-Menten kinetics, dashed lines substrate inhibition kinetics. Substrate inhibition equation: rate = V_max_ × [S]/(Km + [S] + ([S]^2^/K_i_)). Assay methodology is as described in the Materials and methods; in brief, activities were determined by HPLC analysis after a 30-s reaction time. Data points are the mean from three measurements; error bars indicate SDs of three reactions. For parameters, see Table 4.

The apparent kinetic parameters determined in the present study differ considerably from those determined in previous investigations (Fig. 10 and Table 4). For wild-type (varying dopamine with 4-HPAA), we observed an apparent *k*_cat_ and *K*_m_ of 24 s^−1^ and 22 mm respectively, which are substantially larger than the previously determined values of 4.5 s^−1^ and 0.4 mm [16]. Also notable is an absence of cooperativity from the kinetics, with respect to both dopamine and aldehydes (Fig. 10). This is in accordance with the observation that Δ29*Tf*NCS is monomeric at concentrations below 10 μm [21].

The kinetic parameters determined here describe an enzyme that is catalytically inefficient. NCS exhibits an apparent *k*_cat_/*K*_m_ of 1.0 s^−1^·mm^−1^, which is 100-fold below the median of 125 s^−1^·mm^−1^ calculated across all enzymes (Table 4) [25]. The *K*_m,app_ of dopamine with 4-HPAA is 22 mm, showing that high substrate concentrations are required for the enzyme to demonstrate significant turnover. With this in mind, it is expected that NCS operates *in vivo* with substrate concentrations far below a saturating level. For comparison, absolute intracellular metabolite concentrations calculated in *Escherichia coli* showed only three of 103 primary metabolites had concentrations above 10 mm, and over 70% had concentrations below 1 mm [26]. This observed catalytic inefficiency may explain the poor activity of recombinant NCS *in vivo* [10,11].

Plant secondary metabolism enzymes often display low catalytic efficiency [27]. This may be a result of low selective pressure towards optimization because limited flux is required through the pathways. In the case of NCS, the high sensitivity towards substrate concentration, coupled with the fact that the enzyme catalyzes the first committed step of the BIA pathway, indicates that it may be a gatekeeper enzyme and thus be responsible for restricting entry into the BIA pathway. In particular, the high apparent *K*_m_ values would enable control of flux through the pathway by variation of local dopamine, 4-HPAA and NCS concentrations.

### The substitution L76A causes change in aldehyde activity profile

The ability to rationally engineer NCS activity will enable the facile biocatalytic syntheses of diverse (*S*)-THIQs. The dopamine-first mechanism predicts that Leu76 is proximal to the aldehyde R-group (Fig. 4) and so, based on that mechanism, it was hypothesized that amino acid substitutions of Leu76 would show modified activities with different aldehydes. A change in the aldehyde activity profile of a Leu76 variant would validate the dopamine-first mechanism because the aldehyde binding site for the HPAA-first mechanism is in a different location.

The substitution L76A improves the activity of NCS towards both (*S*)- and (*R*)-citronellal but reduces activity towards 4-HPAA and hexanal (Table 4). This change is most notable with (*S*)-citronellal where the apparent *k*_cat_ is doubled both with respect to dopamine and the aldehyde. Interestingly, apparent *K*_m_ values are largely unaffected by the substitution. The data may also suggest that citronellal inhibits the enzyme at high concentrations (Fig. 10 and Table 4), which would be in accordance with the NMR observation that dopamine does not bind to the enzyme subsequent to aldehyde binding [21].

**Fig. 11:**
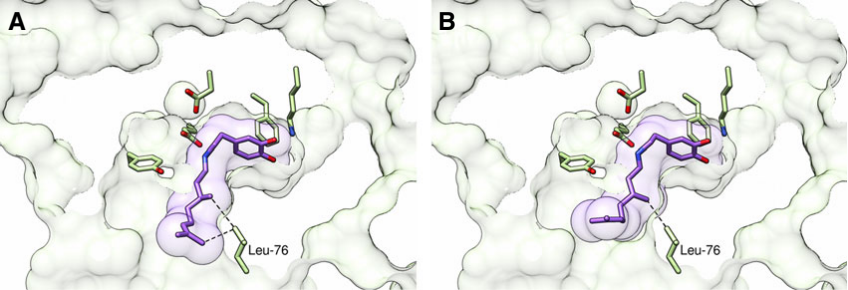
Docked dopamine-citronellal iminium intermediates. (A) (*S*)-citronellal intermediate (highest ranked dock). (B) (*R*)-citronellal intermediate (second highest ranking dock). The intermediates dock in a dopamine-first manner. Note that the methyl groups on the citronellals are close to Leu76. For intermediate structures, see Fig. 7. Reaction intermediates were docked in the NCS active site (subunit A, 2VQ5), using autodock vina. Active site residues carbons are coloured yellow, docked ligands carbons coloured purple. Ligand accessible surfaces are represented by transparent purple. Ligand accessible surfaces are represented by transparent purple. A section of the enzyme solvent accessible surface is represented by transparent green. Images were generated using *Chimera*.

Docking calculations of the imine-citronellal reaction intermediates suggest a molecular origin to this activity increase (Fig. 11). The methyl groups of the (*S*)- and (*R*)-citronellal intermediates are relatively close to Leu76: at 3.65 and 3.75 Å respectively. Removing the steric bulk close to this methyl moiety may enable greater conformational freedom in the intermediate, entropically reducing the reaction energy barrier. The enhancing effect of the amino acid substitution may be more pronounced for (*S*)- rather than (*R*)-citronellal simply as consequence of proximity of the methyl group to Leu76.

The substitution L76A has modified the aldehyde activity profile of NCS. This supports the dopamine-first reaction mechanism and also demonstrates the potential for engineering NCS towards other substrates and activities.

## Conclusions

In the present study, we have described and assessed two different mechanisms of NCS activity: the HPAA-first mechanism and the dopamine-first mechanism. Activities of *Tf*NCS variants appear to support the dopamine-first mechanism. By avoiding the use of phosphate buffer in enzyme assays, we observed novel kinetic parameters that show NCS operates with low catalytic efficiency. This can account for its apparent ineffectiveness in recombinant *in vivo* systems, and may indicate a possible *in planta* role as a BIA gatekeeper enzyme. The amino acid substitution L76A, in the proposed dopamine-first aldehyde binding site, resulted in a change in the aldehyde activity profile, strongly supporting the mechanism in question.

Further experiments are required to fully characterize NCS behaviour both *in vitro* and *in vivo*. For example, double-reciprocal plots with an appropriate inhibitor will provide a kinetic assessment of the compulsory-order binary mechanism proposed in the present study. It may also be possible to monitor the interaction between NCS and substrates via direct methods such as NMR [21]. Future progress with *in planta* proteomics and metabolomics may reveal more details about the involvement of NCS in the control of flux into the BIA pathway [28].

The work reported from the present study has wide implications: not only have we gained insight into the early steps of *in planta* BIA biosynthesis, but also we have demonstrated that it is possible to modify the aldehyde activity profile of NCS via amino acid substitution. Our determination of the NCS mechanism will give rise to the future generation of NCS variants capable of catalyzing the stereoselective formation of diverse THIQs.

## Materials and methods

### Computational methods

#### Molecular Dynamics

Molecular dynamics simulations were conducted in gromacs using the charmm27 forcefield [29,30]. Each subunit of 2VQ5 was prepared by removing waters, ligands and N- and C-termini. The protein subunits were placed in a cube, then water (tip3p) and counter ions were added. The systems were energy minimized (*F*_max_ < 250 kJ·mol^−1^·nm^−1^). This was followed by *NVT* equilibration (100 ps) and *NPT* equilibration (100 ps). Three simulations for each subunit ran for 100 ns, at 300 K. The Phe112 conformation was determined by calculating the angle between the Cα and Cζ of Phe112 and the Cα of His106. A value for this angle was calculated for every tenth frame.

#### Docking

Potential reaction intermediates (Fig. 7) were docked into the active site of subunit A from the *Tf*NCS crystal structure 2VQ5 (Table 1) [16]. Docking was performed using autodock vina (exhaustiveness = 8) [31]. Unless noted otherwise, only the top ranked binding modes were used.

#### p*K*_a_ predictions

propka, version 3.1 [32] was used to predict *p*K_a_ values of residues in the enzyme active site. The *Tf*NCS enzyme structure model was obtained from crystal structure 2VQ5 subunit A [16]. Protein–ligand structures derived from docking calculations (see above) were also analyzed in this manner.

### Protein expression and purification

Genes for N-terminal His-tagged Δ29TfNCS and variants were synthesized and cloned into pJ411 vectors (DNA2.0; Menlo Park, CA, USA) [33]. The plasmids were transformed into BL21(DE3) cells (New England Biolabs, Beverly, MA, USA). The transformed cells were grown in TB media for 16 h at 37 °C with 50 μg·mL^−1^ kanamycin. This overnight culture was transferred into fresh media (4% v/v) and grown at 37 °C for 2 h, then at 25 °C for 1 h. Expression was induced by the addition of isopropyl thio-β-d-galactoside (final concentration: 500 μm) and cells were harvested by centrifugation after 3 h at 25 °C.

Cell pellets were resuspended in Bugbuster (10% culture volume) (Merck Millipore, Darmstadt, Germany) and the insoluble portion removed by centrifugation. The supernatant was passed through Ni-Sepharose HP resin (GE Healthcare, Little Chalfont, UK) equilibrated in binding buffer (20 mm imidazole, 100 mm NaCl, 50 mm Hepes, pH 7.5). The resin was washed with binding buffer (five column volumes) and wash buffer (five column volumes, 40 mm imidazole, 100 mm NaCl, 50 mm Hepes, pH 7.5). NCS was eluted with elution buffer (five column volumes, 500 mm imidazole, 100 mm NaCl, 50 mm Hepes, pH 7.5) and desalted into assay buffer (10% v/v glycerol, 50 mm Hepes, pH 7.5) using PD-10 columns (GE Healthcare). Protein purity was established by SDS/PAGE (12% w/v polyacrylamide). The protein was of sufficient purity for concentration to be determined using *A*_280_. Purified protein was frozen in liquid nitrogen and stored at −80 °C. Enzyme melting and aggregation temperatures were determined using an Avacta Optim 1000 (Avacta Analytical Ltd, York, UK). Runs were performed in triplicate.

### Enzyme assays

#### General

Reactions were conducted using 20 or 80 μg·mL^−1^ of purified enzyme in 50 mm Hepes, with varying concentrations of dopamine and aldehydes, in a total volume of 100 μL. Reaction components were equilibrated at 37 °C and the reaction was started by the addition of enzyme to a substrate mixture. The reaction was incubated at 37 °C and terminated by the addition of 20 μL of 1 m HCl. Reactions were performed in triplicate; all errors are SDs. The background reactions were recorded by performing the reaction in Hepes buffer without enzyme and this was subtracted from the enzymatic reactions.

#### Conversions

Reactions were conducted with 80 μg·mL^−1^ of enzyme, 10 mm dopamine and 10 mm of either 4-HPAA or hexanal. Reactions were performed for 1 h, and were analyzed by analytical and chiral HPLC.

#### Initial rates

Reactions were performed with an enzyme concentration of 20 μg·mL^−1^. Initial rates were determined by measuring the conversion after 30 s, within the linear phase of the reaction. Initial rates were determined with 10 mm of one substrate and 2.5 mm of the other.

#### Kinetics

To determine kinetic parameters, initial rates were measured with varying substrate concentrations. Dopamine concentrations were varied between 500 μm and 20 mm in the presence of 2.5 mm aldehyde. Aldehyde concentrations were varied between 250 μm and 15 mm in the presence of 2.5 mm dopamine. The concentrations of substrates that were not varied during the assays were kept below saturating levels to limit the non-enzymatic reaction. Similarly, higher concentrations of the varying substrates were not used because the increased background reactions led to increased errors. The kinetic data were calculated by fitting initial rates to a Michaelis–Menten or substrate inhibition equation using the nonlinear least-squares analysis in the r software environment (The R Project for Statistical Computing, Vienna, Austria).

#### HPLC analysis

Analyses were performed on a HPLC system consisting of an LC Packing FAMOS Autosampler, a P680 HPLC Pump, a TCC-100 Column oven and a UVD170U Ultraviolet detector (Dionex, Sunnyvale, CA, USA). Quantification of enzyme substrates and products were determined by analytical reverse-phase HPLC with a C18 (150 × 4.6 mm) column (ACE, Aberdeen, UK) and a gradient of H_2_O [0.1% trifluoroacetic acid (TFA)]/MeCN from 9 : 1 to 3 : 7 over 6 min, at a flow rate of 1 mL·min^−1^. The column temperature was 30 °C, and compounds were detection by monitoring *A*_280_. Retention times were 4.9 min and 5.9 min for (*S*)-norcoclaurine and the hexanal product, respectively. The enantiomeric excess (*ee*) was determined by chiral HPLC analysis using a Supelco Astec Chirobiotic T2 (250 × 4.6 mm) column (Sigma-Aldrich, St Louis, MO, USA) and an isocratic MeOH (0.1% TFA, 0.2% triethylamine) mobile phase at 1 mL·min^−1^ and 30 °C. Compounds were detected by monitoring *A*_230_.

### Chemicals

Hexanal, dopamine hydrochloride, (*S*)- and (*R*)-citronellal were purchased from Sigma-Aldrich. 4-HPAA and racemic BIAs for HPLC standards were synthesized as described by Pesnot *et al*. [14]. Characterization data for the previously unreported hexanal product, (1*S*)-1-pentyl-1,2,3,4-tetrahydro-isoquinoline-6,7-diol (TFA salt): ^1^H NMR (500 MHz; CD_3_OD) δ 0.93 [3H, *t*, *J* = 7.1 Hz, (CH_2_)_3_C*H*_3_), 1.38–1.50 (6H, *m*, (C*H*_2_)_3_CH_3_], 1.87 (1H, *m*, CHC*H*H), 2.01 (1H, *m*, CHCH*H*), 2.86–3.00 (2H, *m*, 4-H_2_), 3.29–3.34 (1H, *m*, 3-*H*H), 3.50 (1H, *m*, 3-H*H*), 4.34 (1H, dd, *J* = 8.3 and 5.0 Hz, 1-H), 6.60 (1H, *s*, 5-H), 6.64 (1H, *s*, 8-H); ^13^C NMR (125 MHz; CD_3_OD) δ 14.3, 23.5, 25.7, 26.1, 32.7, 35.1, 41.0, 56.7, 113.9, 116.2, 118.3 (*q*, ^1^*J*_CF_ 295 Hz, CF_3_), 123.7, 124.3, 145.9, 146.7, 163.2 (br, CF_3_CO_2_); *m*/*z* [HRMS ES+] found MH^+^ 236.1639. C_14_H_22_NO_2_ requires 236.1651.

## Abbreviations

4-HPAA: 4-hydroxyphenylacetaldehyde
BIA: benzylisoquinoline alkaloids
*Cj*NCS2: *Coptis japonica*
NCS: norcoclaurine synthase
PHB: *para*-hydroxybenzaldehyde
TFA: trifluoroacetic acid
*Tf*NCS: *Thalictrum flavum*
THIQ: tetrahydroisoquinoline
